# Unveiling the microhabitat puzzle: how spatial heterogeneity shapes cave invertebrate biodiversity across scales

**DOI:** 10.1007/s00442-026-05928-z

**Published:** 2026-06-29

**Authors:** Leandro Mata da Rocha Melo, Rodrigo Lopes Ferreira, Marconi Souza Silva

**Affiliations:** 1 Centro de Estudos Em Biologia Subterrânea, Departamento de Ecologia E Conservação, Instituto de Ciências Naturais, Universidade Federal de Lavras, Campus Universitário, P.O. Box 3037, Lavras, CEP 37200-000 Minas Gerais Brasil; 2 Programa de Pós-Graduação Em Ecologia Aplicada, Universidade Federal de Lavras, Lavras, CEP 37200-000 Minas Gerais Brasil

**Keywords:** Caves, Diversity, Ecology, Gap, Communities

## Abstract

Caves harbor diverse invertebrate communities, but the processes shaping their diversity remain poorly understood in tropical systems. In this study, we tested the hypothesis that physical, trophic, and microclimatic factors drive patterns of species composition, richness, and phylogenetic structure in cave invertebrate communities. To address this, we conducted standardized active sampling of both microhabitat characteristics and invertebrate fauna across multiple spatial scales: cave-level, mesoscale (3 × 10 m), and microscale (1 × 1 m). We recorded a total of 357 invertebrate species, representing at least 55 orders, including 28 troglomorphic taxa. At the microscale, species composition was influenced by distance from the entrance as well as by spatial separation among caves, indicating that dispersal limitation, coupled with environmental constraints such as oligotrophy in deeper zones, acts as an important structuring process. At the mesoscale, variation in shelter diversity and temperature was associated with species composition, suggesting that structural heterogeneity and microclimatic gradients function synergistically as environmental filters. Species richness at the microscale was positively related to both distance from the entrance and the availability of trophic resources, highlighting the role of resource input in promoting colonization, particularly in entrance zones. Phylogenetic distance was influenced by distance from the cave entrance and by trophic resource diversity. Collectively, these results provide mechanistic insights into the assembly of cave invertebrate communities and contribute to the development of conservation strategies tailored to karst ecosystems.

**Supplementary Information:**

The online version contains supplementary material available at 10.1007/s00442-026-05928-z.

## Introduction

One of the central challenges in ecological research on subterranean environments is to understand how environmental factors shape community diversity and composition across different spatial scales (Laliberté et al. 2009). Regional diversity is largely governed by broad-scale evolutionary and historical processes, including speciation, extinction, and long-term biogeographic dynamics (Ricklefs 2004; Cornell and Harrison 2014). By contrast, local diversity is more strongly determined by ecological mechanisms, particularly environmental filtering (which favors species with traits adapted to prevailing conditions) and dispersal barriers, which constrain the arrival of potential colonizers (Vellend 2010; Mammola and Isaia 2018). Collectively, these processes underscore the interplay of scale-dependent drivers that structure biodiversity patterns (da Costa et al. 2019).

A complex interplay of evolutionary and historical processes also shapes regional species diversity in cave ecosystems. These factors, including the geological history of karst landscapes and the long-term isolation of cave habitats, contribute to the overall diversity observed across regions (Van Hengstum et al. 2019; Vaccarelli et al. 2023). At the local scale, however, diversity within individual caves is shaped predominantly by ecological mechanisms, especially environmental filtering and dispersal barriers (Mammola and Isaia 2018; Souza-Silva et al. 2021). Environmental filtering favors species capable of persisting under the characteristic microclimatic and physical conditions of caves, including stable temperatures, high humidity, and low nutrient availability (Culver and Pipan 2019). Dispersal barriers, such as lithological discontinuities and the limited connectivity of subterranean systems, further restrict species movement and promote distinct community assemblages (Marsh et al. 2023). Together, these processes underscore the importance of both broad-scale historical influences and fine-scale habitat characteristics in shaping cave invertebrate communities (Souza-Silva et al. 2021; Vaz et al. 2025).

Recent research has demonstrated that the richness and composition of subterranean invertebrate communities are closely linked to multiple environmental factors, including distance from the cave entrance, horizontal and vertical spatial gradients within caves, habitat heterogeneity, resource availability, microclimatic conditions, lithology, and the presence of aquatic habitats such as water puddles, phreatic lakes, and both allogenic and autogenic streams (Mammola et al. 2020a, b; Mazebedi and Hesselberg 2020; Balestra et al. 2021; Knight et al. 2022). The influence of these factors, however, is not uniform across spatial scales. Patterns evident at the microhabitat level may differ markedly from those detected at the scale of individual caves or entire cave systems within a karst landscape (Pacheco et al. 2020; Vaz et al. 2025). Consequently, an explicit consideration of scale is fundamental for accurately interpreting faunal responses to environmental gradients.

Other variables such as ecological interactions, phylogenetic distance, and biological invasion, which may play important roles in shaping fauna, have been largely neglected in ecological studies of cave environments (da Rocha Melo et al. 2025a). Nevertheless, the integration of phylogenetic information into ecological research has gained increasing relevance, expanding our understanding of biodiversity patterns and community assembly processes (Tucker et al. 2017). In cave ecology, this approach may enable investigations into how mechanisms such as habitat filtering, competition, and coexistence dynamics influence phylogenetic clustering or overdispersion among species (Oliveira and Ferreira 2024).

Environmental factors can thus act as filters, determining which species can colonize, persist, and coexist in certain microhabitats (Culver and Pipan 2019). In this process, they may promote niche specialization, spatial segregation, and physiological adaptations to oligotrophic conditions and the environmental stability typical of caves. The persistence of these organisms depends on their ability to exploit specific niches, reduce direct competition, and efficiently utilize limited resources (Schneider et al. 2011; Mammola et al. 2016). In deeper zones permanently deprived of light, such selective pressures may favor the occurrence of highly specialized species restricted to subterranean life, potentially resulting in the convergence of morphological traits even among distantly related taxa (Krishnan and Rohner 2017; Sumner-Rooney 2018). This process may lead to the development of troglomorphisms, morphological modifications associated with the subterranean lifestyle, such as eye reduction or loss, elongation of appendages, and depigmentation (Christiansen 1962). These morphological, physiological, and behavioral traits are directly linked to the evolutionary trajectory of subterranean invertebrates and are used to classify them into different categories according to their relationship with the environment: trogloxenes (species that use caves but are not restricted to them), troglophiles (species able to inhabit both surface and subterranean environments), and troglobites (species strictly specialized for cave life, mainly recognized through troglomorphisms) (Schiner 1854; Racovitza 1907; Howarth 1983; Sket 2008). It is important to emphasize that, at the global scale, the majority of cave-dwelling organisms are assigned to the first two ecological categories, whereas troglobites constitute a comparatively small fraction of subterranean fauna and are typically rare and highly endemic (Sket 2008; Culver and Pipan 2019). Nevertheless, the behavioral ecology of many hypogean taxa remains insufficiently understood, which can complicate their accurate classification within this framework. For instance, occasional records of surface activity in taxa traditionally regarded as strictly troglobitic, such as *Proteus anguinus*, highlight conceptual ambiguities and limitations inherent to the categorical nomenclature commonly used in subterranean biology (Manenti et al. 2024; Barzaghi et al. 2025).

It is well established that both natural and anthropogenic disturbances in cave environments can alter microhabitat structure and, consequently, affect their associated fauna. Natural disturbances include events such as roof collapses and hydrological fluctuations, whereas anthropogenic pressures are primarily linked to urban expansion, mining, agriculture, uncontrolled tourism, and deforestation in the surroundings of caves (Souza-Silva et al. 2015; Mammola et al. 2019; Da Rocha Melo et al. 2025b). Karst regions containing caves are widely distributed across the globe, yet they have been heavily impacted, particularly by urban expansion, mining, and agricultural activities (Jaffé et al. 2018; Ferreira et al. 2022). Such pressures can result in the partial or complete destruction of caves, as well as alterations to their surroundings, thereby modifying the quality and quantity of organic and inorganic substrates entering the subterranean environment (Cardoso et al. 2022). Because cave ecosystems are highly dependent on external resource input and characterized by long-term environmental stability, their ecological integrity is especially vulnerable to disturbance. Disruptions of this kind can have cascading effects on the composition, abundance, and persistence of invertebrate communities inhabiting these systems (Ferreira and Horta 2001; Souza-Silva et al. 2015). Troglobitic species are particularly susceptible, as they frequently exhibit narrow ranges of distribution and high levels of endemism, often being restricted to a single region or even a single cave (Sket 2008; Culver and Pipan 2019; Junta et al. 2025).

Given this context, the present study aimed to evaluate the influence of trophic, physical, and microclimatic factors on the structure of invertebrate communities in caves located within three limestone ranges of the Brazilian Atlantic Forest: Capiru, Itaiacoca, and Votuverava. More specifically, we sought to determine how these environmental variables operate across different spatial scales and to identify the mechanisms regulating species richness and composition. To this end, we tested the following hypotheses: (i) species composition differs among karst regions as a result of limited connectivity and geological barriers that restrict invertebrate dispersal; (ii) species richness and composition are structured by environmental filtering, with higher diversity associated with increased resource availability and habitat heterogeneity; and (iii) phylogenetic distance in cave communities is modulated by environmental factors, such that greater resource diversity and habitat heterogeneity promote the coexistence of more phylogenetically distinct lineages, whereas restrictive conditions favor clustering among closely related taxa.

## Methodology

### Study area

**Fig. 1:**
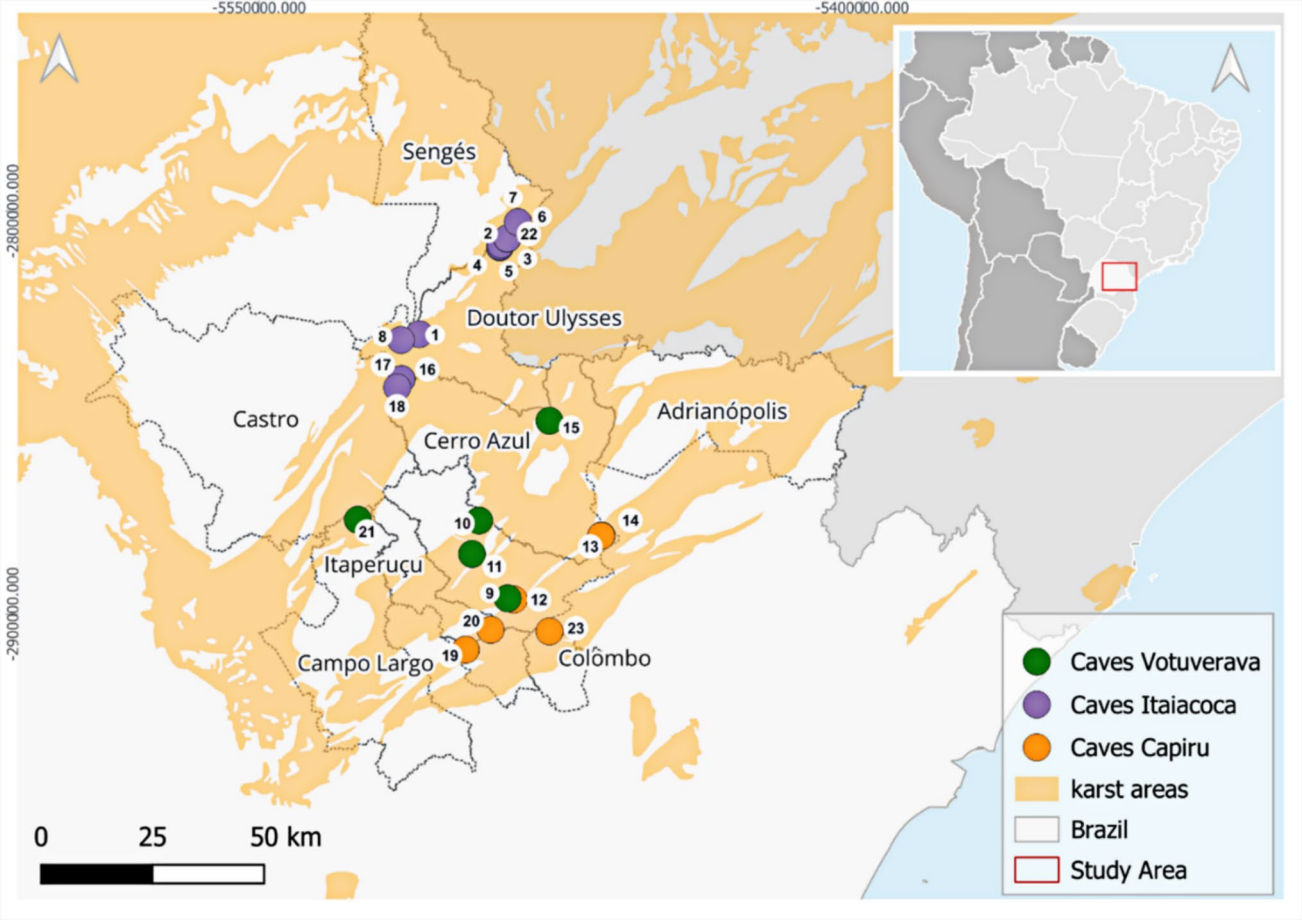
The municipalities are located in the metropolitan region of Curitiba, Paraná state, Brazil, and the studied caves. The circles represent the limestone regions Votuverava (purple), Itaiacoca (green), and Capiru (Orange). The numbers represent the caves studied. 1-Varzeão cave; 2-Casa de Pedra cave; 3-Ressurgência do Feital cave; 4-Dá a volta cave; 5-Arco de Pedra cave; 6-Pinhalzinho cave; 7-Pocinho cave; 8-Malfazido cave; 9-Toquinhas cave; 10-Piedade cave; 11-Bromados cave; 12-Lancinha cave; 13-Fadas cave; 14-Jesuítas cave; 15-Bom Sucesso cave; 16-Chiquinho cave; 17-Chiquinho II cave; 18-Pinheiro Seco cave; 19-Ermida cave; 20-Itaperussu cave; 21-Pinheirinho cave; 22-Pocinho II cave; 23-Bacaetava cave

This study was conducted in caves located within three limestone regions (Capiru, Votuverava, and Itaiacoca) in the northern part of Paraná State, Brazil. These regions encompass the municipalities of Colombo, Sengés, Castro, Campo Largo, Rio Branco do Sul, Almirante Tamandaré, Itaperuçu, Cerro Azul, Adrianópolis, and Doutor Ulysses (Fig. 1). The limestone formations of Capiru, Votuverava, and Itaiacoca are composed of metasedimentary rocks belonging to the Açungui Carbonate Group. The region is characterized by a humid subtropical climate, with mild winters and evenly distributed rainfall throughout the year. It lies within the Atlantic Forest biome and encompasses both Araucaria Forest (Mixed Ombrophilous Forest) and Dense Ombrophilous Forest (the proper Atlantic Rainforest) (Passos 1986).

Several proposals have been made over the years regarding the delimitation of these karst regions (Almeida 1957; Fiori et al. 1992; Reis Neto 1994). In this study, we adopt the regionalization proposed by Sessegolo et al. (2006), in which the Capiru Region includes all metasedimentary units of the Açungui Group located south of the Lancinha Fault, at the base of the carbonate sequence. The Votuverava region, composed of carbonate rocks, is situated north of the Lancinha Fault, between the Capiru and Itaiacoca regions. The Itaiacoca Region lies between the Serra de Itaiacoca in Paraná and the Taquari Mirim River Valley in São Paulo State. This area comprises a complex of metavolcanic and metasedimentary rocks, for which several stratigraphic frameworks have been proposed (Almeida 1957; Trein et al. 1985; Reis Neto 1994).

Twenty-three caves were sampled, twelve of these in the Itaiacoca limestone region, five caves located in the Votuverava limestone region, and another six caves situated in the Capiru limestone region (Fig. 1, Table 1).

**Table 1:** Caves sampled in the Curitiba metropolitan region, Paraná, Brazil (cave name). Limestone regions (Region), latitude (Lat), and longitude (Long) in UTM, the number of sectors (NS), and quadrats (NQ). Itaiacoca (ITA), Votuverava (VOT), Capiru (CAP), regions. (LN) Linear development

Cave name	Belt	Lat	Long	NS	NQ	LN(m)	Touristic
Varzeão	ITA	7275042	651909	8	24	2087	No
Casa de Pedra	ITA	7294947	670668	1	3	30	No
Ress. do Feital	ITA	7294326	670403	4	12	327	No
Dá a volta	ITA	7294572	670427	2	6	2675	No
Arco de Pedra	ITA	7294581	670419	1	3	30	No
Pinhalzinho	ITA	7299940	674573	5	15	914	No
Pocinho	ITA	7296230	672123	4	12	625	No
Malfazido	ITA	7273761	647782	4	12	631	No
Toquinhas	VOT	7215636	671190	3	9	NA	No
Piedade	VOT	7233100	664985	3	9	NA	No
Bromados	VOT	7225641	663298	4	12	704	No
Lancinha	CAP	7215358	672566	4	12	2080	No
Fadas	CAP	7229326	692470	1	3	1565	Yes
Jesuítas	CAP	7229413	692528	4	12	1565	Yes
Bom Sucesso	VOT	7255243	681136	3	9	NA	No
Chiquinho	ITA	7264965	647839	2	6	NA	No
Chiquinho II	ITA	7264954	647825	3	9	NA	No
Pinheiro Seco	ITA	7263142	646809	6	18	650	No
Ermida	CAP	7204278	661607	2	6	297	No
Itaperussu	CAP	7208651	667277	4	12	570	No
Pinheirinho	VOT	7233572	637641	5	15	1580	No
Pocinho II	ITA	7296306	672130	1	3	NA	No
Bacaetava	CAP	7208130	680519	6	18	695	Yes

### Measuring microhabitat features on the cave floor

**Fig. 2:**
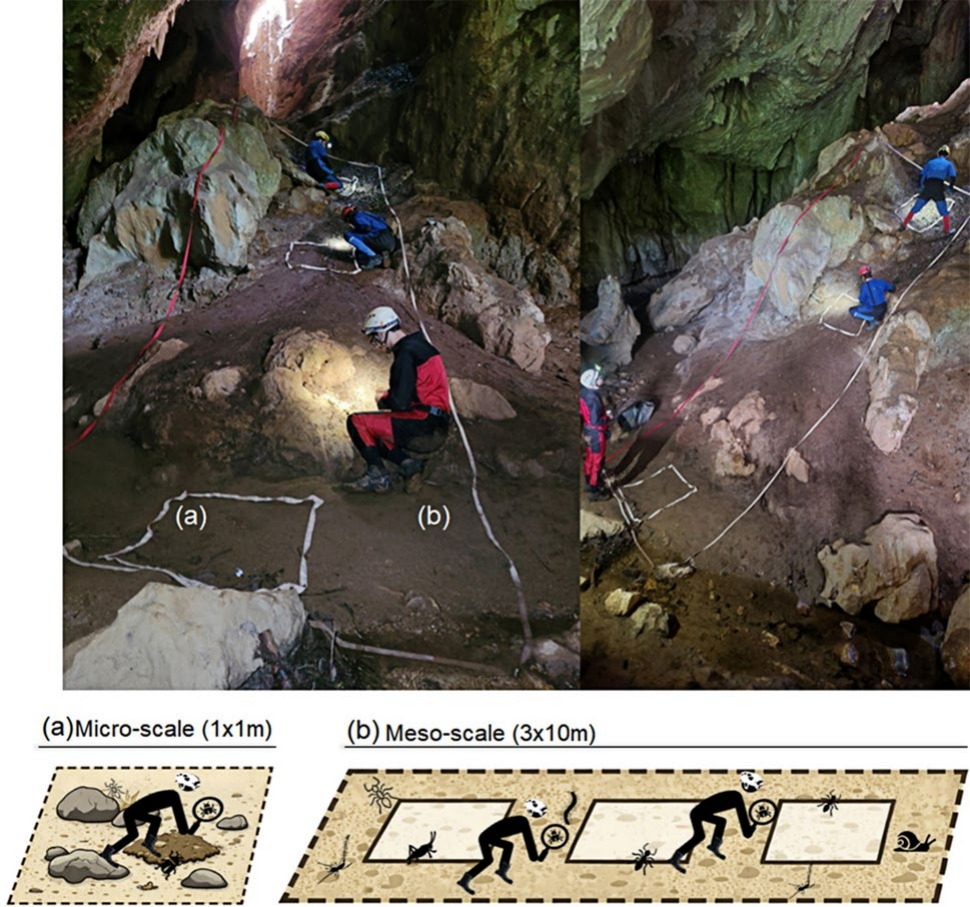
Sampling of cave invertebrates at different spatial scales inside the caves. (**a**) Microscale sampling using 1 × 1 m quadrats and (**b**) mesoscale sampling using 3 × 10 m sectors. Invertebrates were collected manually with tweezers and brushes and preserved in containers with 70% ethanol. Photographs taken in Jesuítas Cave, Cerro Azul, Capiru limestone region, southern Brazil

Microhabitat structure on the cave floor was characterized through visual inspection and quantification of the surface area occupied by distinct organic and inorganic components within sectors and quadrats as established by Pacheco et al. (2020) and Souza-Silva et al. (2021) (Fig. 2). Sampling efforts were focused on the cave floor, as this microhabitat exhibits greater substrate heterogeneity and structural complexity, thereby providing higher potential for testing our hypotheses. Although differences in community composition among floor, wall, and ceiling habitats have been documented (Balestra et al. 2021), logistical and temporal constraints precluded the systematic sampling of vertical surfaces. Nevertheless, the cave floor is widely recognized as harboring the highest diversity of invertebrates, as it functions as the primary compartment for resource accumulation and trophic support in subterranean environments (Souza-Silva et al. 2021; Vaz et al. 2025). This ecological role further justifies our emphasis on this microhabitat.

Floor substrates were classified into three main categories: trophic resources, shelter resources, and a mixture of both (general microhabitats) (Table S1). Specifically, each sector was subdivided along its longest axis into ten perpendicular sections, each measuring 1 × 3 m. The percentage of area occupied by components of the substrate elements (both organic and inorganic) was accounted within these sections in terms of proportion. To obtain a final value for each substrate type within the hole sector, the proportional values of each section (Souza-Silva et al. 2021).

Inside quadrats, substrate composition was assessed using in situ photographic documentation. Vertical digital photographs (4000 × 3000 pixels) were captured in the field using a Canon Powershot SX50 HS camera positioned at a 90° angle. These images were analyzed using ImageJ software (Rasband 1997), which allowed for the calculation of the surface area occupied by each substrate component based on pixel proportion (Pacheco et al. 2020). Additionally, microclimatic conditions (temperature and humidity) were measured in each sector using a digital thermometer and hygrometer (AKSO AK-625, with an accuracy of ± 0.8 °C and ± 4% relative humidity) placed directly on the cave floor and kept at a minimum distance of 3 m from the operators to avoid any influence on the readings. Measurements were recorded after a minimum stabilization period of fifteen minutes (Souza-Silva et al. 2021).

### Measuring large-scale environmental factors

As regional scale variables the geographic distance between caves was determined by recording the geographical coordinates of the cave entrances using a Garmin 64 s GPS. QGIS Development Team (2024), Geographic Information System, version 3.34 was used to plot the cave and measure both the distance among caves and their proximity to mining areas.

### Accessing of invertebrates richness and composition

Invertebrate was collected within sectors (3 × 10 m) and quadrats (1 × 1 m) using the Direct Intuitive Search (DIS) method (Pacheco et al. 2020), combined with active collection using forceps and brushes moistened with 70% ethanol, the search time across sampling units ranged from 20 to 40 min, reflecting the substantial variation in substrate conditions among different cave areas (Fig. 2). A total of 80 sectors and 240 quadrats were and established inside 23 caves. The number of sectors per cave varied according to the physical characteristics and spatial extent of each cavity (Table 1). All collections were performed by experienced cave ecologists with experience in cave fauna surveys, following the recommendations of Souza-Silva et al. (2011). Field campaigns were conducted once per cave, spanning three distinct sampling periods: November 2022, April 2023, and July 2023. Although sampling was conducted during different periods of the year, the study region is classified as humid subtropical (Cfa and Cfb, Köppen), characterized by high and relatively evenly distributed precipitation and the absence of pronounced seasonal variation (Álvares et al. 2014). Under these climatic conditions, seasonal influences on cave microclimate and resource inputs are expected to be limited, thereby reducing potential biases associated with sampling across different seasons.

### Sorting and identification of invertebrates

All invertebrate specimens were collected and preserved in vials containing 70% ethanol for subsequent identification and separation into morphotypes (Oliver and Beattie 1996; Furtado-Oliveira et al. 2022). Identification was carried out using available taxonomic keys (Edgecombe 2004; Rafael et al. 2012; Asenjo et al. 2013; Zampaulo and Prous 2022), and specialists confirmed the classification of Acari, Isopoda, Hemiptera, Palpigradi, Pseudoscorpiones, Diplopoda, and Orthoptera (see Acknowledgments). A morphospecies-based approach was adopted rather than assigning specimens to finer taxonomic levels (e.g., genus or species). This decision reflects the taxonomic challenges commonly associated with subterranean invertebrates (Pacheco et al. 2025), as well as the limited availability of specialists for the groups recorded. All samples are deposited in the Cave Invertebrate Collection of Lavras (ISLA), housed at the Center for Studies in Subterranean Biology (CEBS), Federal University of Lavras (UFLA) (biologiasubterranea.com.br).

### Determining invertebrate species with troglomorphic traits

The identification of potentially troglobitic species was based on the presence of troglomorphic traits, which serve as indicators of isolation and evolutionary adaptation to cave environments. Commonly observed troglomorphisms included the reduction or complete loss of ocular structures and pigmentation and the elongation of sensory and locomotor appendages (Table S2) (Culver and Pipan 2019).

### Data analysis

#### Microhabitat structure

Substrate categories listed in Table SI were used to calculate overall substrate diversity, shelter diversity, and trophic resource diversity based on the Shannon–Weaver diversity index (Buttigieg and Ramette 2014). Shelter availability for invertebrates was estimated as the sum of the surface areas occupied by coarse gravel, rock blocks, and organic debris within each transect. Likewise, trophic resource availability was quantified by summing the surface areas corresponding to guano, feces, roots, organic debris, cryptogams, phanerogams, algae, actinomycetes, basidiomycetes, animal carcasses, and pteridophytes.

To assess the influence of distance from the cave entrance on substrate diversity and availability (including shelter and trophic resources), Spearman’s rank correlation analyses were performed, using quadrats and sectors as sampling units and analyzing each karst region separately (Gallucci 2019).

To assess whether substrate composition, substrate diversity, shelter diversity, and trophic diversity differed among limestone regions and between caves, we performed an Analysis of Similarities (ANOSIM) based on Euclidean distance, using sectors and quadrats as independent sampling units (Clarke et al. 2001; Buttigieg and Ramette 2014). This non-parametric method was selected because it is appropriate for testing group differences using similarity matrices (Clarke et al. 2001). Prior to analysis, data were square-root transformed to minimize the influence of extreme values. Results were visualized using shade plots, and a cluster analysis based on the Whittaker Association Index was also applied to identify patterns of substrate co-occurrence (Whittaker 1952; Clarke et al. 2014).

### Variations in species composition

Variations in species composition of invertebrate cave fauna was assessed using the Bray–Curtis similarity index and visualized through non-metric multidimensional scaling (nMDS), with resampling via the bootstrap method. Analysis of Similarities (ANOSIM) based on the Bray–Curtis index was employed to test for compositional differences among carbonate belts, using cave sectors and quadrats as sampling units (Anderson and Santana 2015).

To evaluate the influence of substrate diversity, shelter diversity, trophic diversity, temperature, humidity, geographical distance between caves, distance from the cave entrance, and distance from mining operations on species composition (based on Bray–Curtis similarity), we applied a distance-based linear model (DistLM) using a forward stepwise selection procedure and the corrected Akaike Information Criterion (AICc) for model selection (Anderson and Santana 2015). This method was chosen because it allows direct relationships between similarity matrices and environmental variables, quantifying the relative contribution of each predictor. Subsequently, distance-based redundancy analysis (dbRDA) was used to assess the model’s explanatory power and visualize the proportion of variation explained (Clarke et al. 2006). These analyses were conducted independently for sectors and quadrats (Pacheco et al. 2020).

Invertebrate abundance and richness (alpha diversity) were calculated based on counts of individuals and morphotypes per sampling unit. Individual-based rarefaction curves were generated to estimate expected species richness in each carbonate belt, analyzed separately for quadrats and sectors (Chao and Jost 2012).

### Variations in species richness

To investigate the environmental factors associated with species richness at the mesoscale (sectors), we employed Generalized Linear Mixed Models (GLMMs). This approach was chosen because it is appropriate for count data (species richness) and allows the inclusion of random effects (here, cave), thereby controlling for pseudo replication and structural differences among caves. Analyses were performed in R using the packages lme4 (Bates et al. 2015), DHARMa (Hartig 2016), and performance (Lüdecke et al. 2021).

All predictor variables were standardized (mean = 0, standard deviation = 1) to facilitate comparability among coefficients and improve numerical stability. The predictor set included air relative humidity, air temperature, distance from the cave entrance, trophic diversity, shelter diversity, overall substrate diversity, resource availability, and shelter availability.

Two models were fitted, using the Poisson and negative binomial families, both of which included cave as a random effect. These distributions were tested because richness data often show overdispersion, requiring alternatives beyond the Poisson. Model fit was assessed by simulating residuals (function simulateResiduals from the DHARMa package) to check for systematic patterns not captured by the model. Collinearity among predictors was assessed using check_collinearity (from the performance package), and redundant variables were excluded. Model quality was compared using the likelihood ratio test (anova) between nested models, and marginal R^2^ (variance explained by fixed effects) and conditional R^2^ (fixed + random effects) were obtained using the MuMIn package (Bartoń 2022).

At the microscale (quadrats), we adopted the same approach but included both cave and sector as random effects. The predictors considered were trophic diversity, overall substrate diversity, distance from the entrance, shelter diversity, shelter availability, and resource availability. At the mesoscale, all predictors were standardized (mean = 0, standard deviation = 1). Poisson and negative binomial distributions were again fitted, with overdispersion assessed and model performance compared using AIC. This strategy ensured methodological consistency across scales, allowing direct comparisons.

### Phylogenetic distance

A combination of statistical and ordination analyses was employed to evaluate the phylogenetic distance (distinctness) among cave invertebrate communities and to assess the influence of environmental variables, including relative humidity, air temperature, distance from the cave entrance, trophic diversity, shelter diversity, overall substrate diversity, resource availability, and shelter availability. Prior to model fitting, we evaluated collinearity among distance from the entrance (DE), microclimatic variables (humidity and temperature), and descriptors of habitat and resource heterogeneity. Collinearity was assessed by calculating the Variance Inflation Factor (VIF) using linear models that simultaneously included all environmental predictors. Temperature exhibited strong collinearity with humidity (VIF > 30) and was therefore excluded from subsequent analyses.

Phylogenetic distance was estimated by transforming hierarchical taxonomic identities into a clustering-based representation that quantified dissimilarity across taxonomic levels. This approach generated a taxonomy-based surrogate phylogenetic tree, which served as the backbone for all subsequent phylogenetic distance calculations. Distances were then normalized by the total number of taxonomic levels, following the method proposed by Clarke and Warwick (1999). Phylogenetic distance between community pairs was calculated using the *comdist* function from the *picante* package in R. Species occurrence data were organized as presence–absence matrices (binary coding: 0 = absence, 1 = presence).

To visualize community similarity patterns, Principal Coordinates Analysis (PCoA) was conducted using a Jaccard distance matrix generated via the vegdist function from the vegan package. Environmental variables were explored in relation to phylogenetic distance using generalized linear models (GLMs), followed by segmented regression to identify potential threshold patterns or changes in slope in the relationship between pairwise phylogenetic distance and environmental differences at both micro- and mesoscales. Model performance was evaluated using the corrected Akaike Information Criterion (AICc), and interpretation focused on identifying general patterns and potential breakpoints. Because these analyses are based on pairwise comparisons between communities, involving non-independent observations, they were used for exploratory purposes only and interpreted with caution (Oliveira and Ferreira 2024).

To quantify the independent and shared contributions of spatial position and environmental variables to the phylogenetic structure of communities, we conducted a distance-based redundancy analysis (dbRDA). The phylogenetic distance matrix was used as the response variable, while the same set of predictors employed in the previous analyses was included at both meso- and micro-scales. All dbRDA models were fitted using the *capscale* function in the *vegan* package, and statistical significance was evaluated using permutation tests with 999 permutations.

## Results

### Microhabitat structure on the caves floors

The cave floor substrates are detailed in Table SI and Fig. S1, which include their abbreviations and classification into three categories: trophic (resource), shelter, and general substrates. An Analysis of Similarities (ANOSIM), based on Euclidean distance, revealed no significant differences in substrate composition among the limestone regions, at both the sector and quadrat scales. However, a significant relationship was found between substrate composition and distance from the entrance in the Votuverava and Itaiacoca regions, with floor substrates becoming more homogeneous as the distance from the entrance increased. This pattern was not observed in the Capiru region (Fig. S1).

In the sector-level analysis, certain substrate elements, such as hardpan, guano, speleothems, and large boulders, were more commonly associated with areas farther from the cave entrance (Fig. S1A). In contrast, elements such as animal carcasses, snail shells, algae, wood fragments, and basidiomycetes were more frequently found near cave entrances (Fig. S1A). At the quadrat level, guano, hardpan, gravel, and rough rock were more prevalent in deeper cave areas, whereas wood fragments, basidiomycetes, animal carcasses, and sloped floors were concentrated near the entrances (Fig. S1B).

Trophic and shelter diversity decreased progressively with increasing distance from the cave entrance across all studied regions (Fig. S2). This trend is also evident in the availability of resources and shelter, which declined gradually toward the inner cave zones (Fig. S2). These results indicate that cave entrance zones are characterized by greater microhabitat heterogeneity, while deeper cave areas tend to be more environmentally homogeneous.

### Variations in cave fauna composition

Species composition within quadrats differed significantly between the Votuverava and Capiru formations (Global R = 0.10, p = 0.01), while the highest similarity was observed between Itaiacoca and Votuverava, followed by Itaiacoca and Capiru. A similar pattern was found at the sector level, with Itaiacoca and Votuverava again showing the greatest similarity, followed by Itaiacoca and Capiru. Significant compositional differences between Votuverava and Capiru were also detected at this scale, with a global R-value of 0.14 and p = 0.01.

ANOSIM analysis at the cave level indicated significant differences in species composition across caves (Global R = 0.23, p = 0.011). However, when caves were grouped by limestone region, no significant differences in overall faunal composition were detected. The dispersion patterns of similarity for quadrats and sectors, are illustrated in Fig. S3.

Identified shelter diversity and temperature as significant predictors of species composition at the sector level (AICc = 661.9; R^2^ = 0.06; p = 0.04). At the quadrat level, distance from the cave entrance and geographic distance between caves significantly influenced faunal composition (AICc = 1685.6; R^2^ = 0.025; p = 0.001). Distance-based Redundancy Analysis (dbRDA) for the sector data showed a model fit of 46%, with 9% of the variation explained by the environmental variables tested. For the quadrats, indicated a higher overall model fit of 54.9%, though only 3.5% of the faunal variation was explained by the predictor variables.

### Variations in species richness

**Fig. 3:**
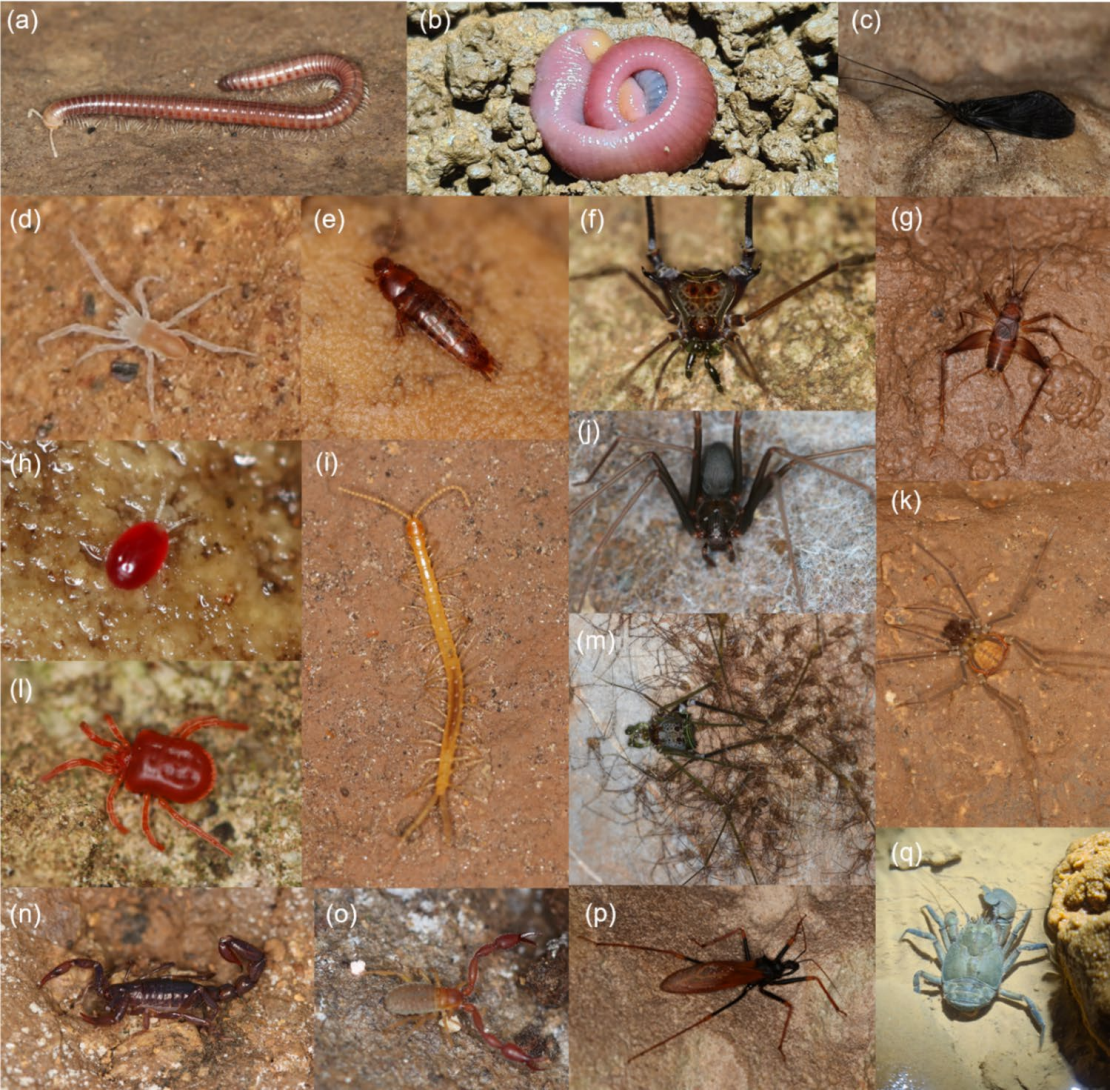
Non-troglobite invertebrates collected in 23 caves from three carbonate bands in the metropolitan region of Curitiba, Paraná. (**A**) *Pseudonannolene* sp. (**B**) Hirudinida sp. (**C**) Trichoptera sp. (**D**) Rhagidiidae sp1. (**E**) Staphylinidae sp. (**F**) *Serracutisoma* sp1. (**G**) *Endecous* sp. (**H**) Macronyssidae sp. (**I**) *Cryptops* sp. (**J**) *Loxosceles* sp. (**K**) Gonyleptidae sp2. (**L**) Trombidiformes sp. (**M**) Gonyleptidae sp3. (**N**) *Thestylus aurantiurus* (**O**) *Spelaeochernes* sp. (**P**) *Zelurus travassosi*. (**Q**) *Aegla* sp

Invertebrate fauna was recorded in 200 quadrats, while 40 quadrats (17%) yielded no invertebrate specimens. The survey identified 357 invertebrate species, representing at least 55 distinct taxonomic orders (see Supplementary Material), with selected representatives illustrated in Fig. 3. Among the three limestone regions, the Capiru formation exhibited the highest average species richness (mean = 53.8, SD = 20.6), followed by Itaiacoca (mean = 52.6, SD = 19.8), and Votuverava (mean = 48.2, SD = 21.9). These averages include invertebrates collected from sectors, quadrats, and general cave sampling. Across all scales, the orders Araneae, Diptera, and Acari stood out as the most species-rich groups (Fig. S4).

Regarding fauna with troglomorphic traits, 28 species have been identified: Four species of Palpigradi: (Eukoeneniidae); Two of Pseudoscorpiones: (Chtoniidae); Four of Opiliones: (Gonyleptidae and Cryptogeobiidae); Three of Araneae: (Hahniidae and Prodidomidae); Seven of Collembola: (Poduromorpha, Entomobryomorpha, and Symphypleona) Three of Isopoda: (Philosciidae and Plathyarthridae); Four of Diplopoda: (Pyrgodesmidae and Oniscodesmidae); and one of Stylommatophora: (Systrophiidae).

**Fig. 4:**
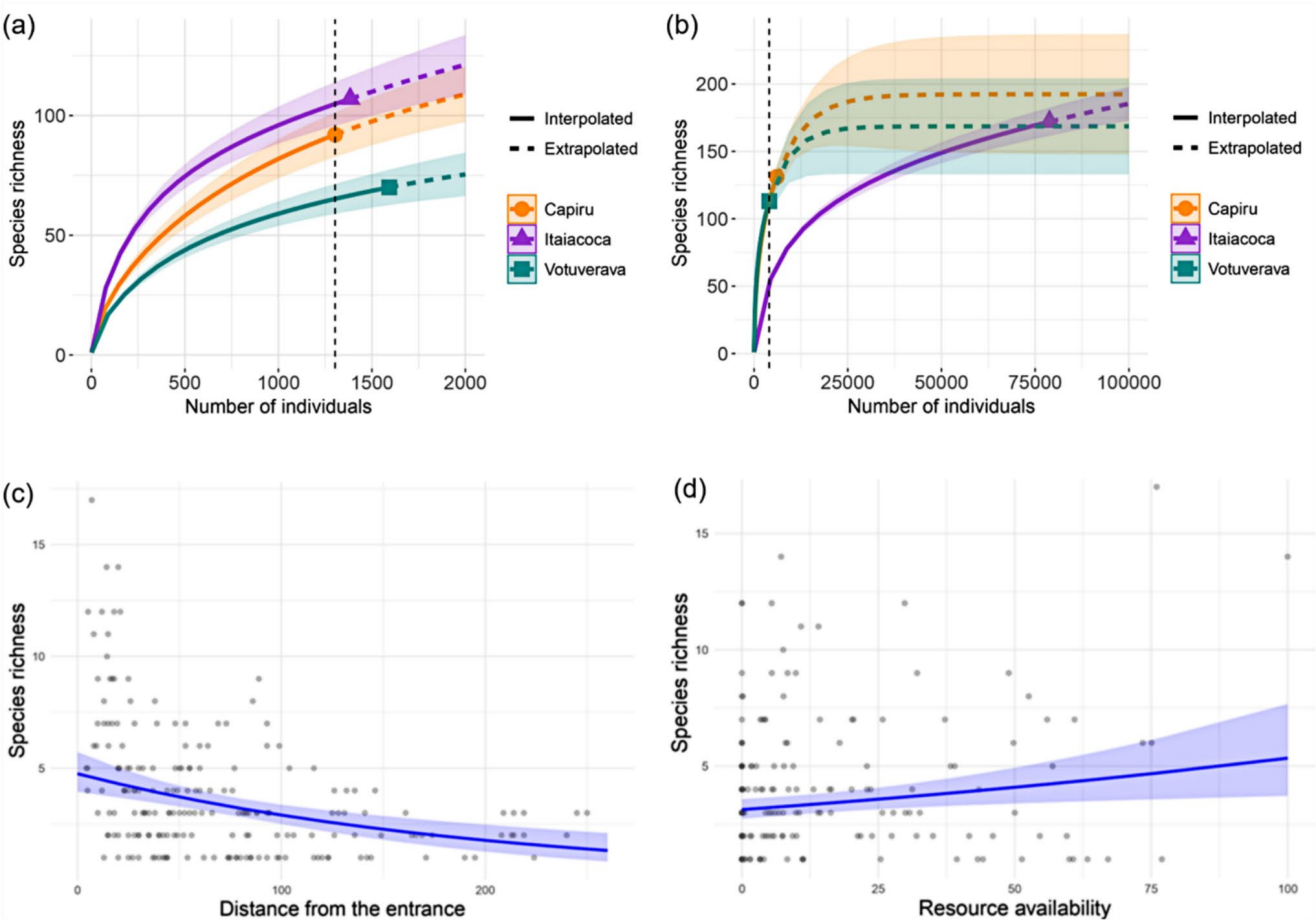
Rarefaction curves based on individuals. (**A**) Rarefaction curve performed on a microscale (quadrants) (**B**) Rarefaction curve performed on a mesoscale (sectors). The black dashed line represents the community with the lowest abundance, the continuous colored lines represent the richness and abundance of collected species, and the dashed lines represent the extrapolation of the data. The relationship between the species richness and distance from the entrance (4**C**) and availability of resources on a micro scale (**D**)

Individual-based rarefaction curves, calculated separately for sectors and quadrats within each limestone belts, suggest that species richness in sectors is likely underestimated, particularly in the Capiru and Votuverava, indicating the potential for new species discoveries with additional sampling. In contrast, at the microscale, species richness observed in quadrats closely matched the extrapolated values across all three limestone belts, with narrow confidence intervals suggesting sampling sufficiency (Fig. 4A; Fig. 4B).

At the microscale, the model assessing species richness revealed a significant positive relationship with resource availability and a significant negative relationship with distance from the cave entrance (Table 2; Fig. 4C; Fig. 4D). In contrast, none of the environmental variables tested at the mesoscale showed a significant association with species richness (Table 2).

**Table 2:** Summary of generalized linear mixed models (GLMMs) evaluating the effects of environmental predictors on cave invertebrate species richness at two spatial scales: microscale (quadrant level) and mesoscale (sector level)

Spatial scale	Variable	Estimate	Std. Error	Z value	p-value
**Microscale**	(Intercept)	1.212742	0.064327	18.853	< 2e-16 *
**1 × 1 m**	Trophic diversity	0.038196	0.038175	1.001	0.31705
	General substrate diversity	0.002658	0.051017	0.052	0.95846
	Distance from the entrance	-0.280004	0.065671	-4.264	2.01e-05 *
	Shelter diversity	0.034861	0.060734	0.574	0.56597
	Shelter availability	0.065556	0.048331	1.356	0.17498
	Resource availability	0.106641	0.040291	2.647	0.00813 *
**Mesoscale**	(Intercept)	2.619899	0.091201	28.727	< 2e-16 *
**3 × 10 m**	Humidity	0.000238	0.094092	0.003	0.998
	Temperature	-0.039931	0.085596	-0.467	0.641
	Distance from the entrance	0.035589	0.084242	0.422	0.673
	Trophic diversity	0.107834	0.090542	1.191	0.234
	Shelter diversity	0.082252	0.085880	0.958	0.338
	General substrate diversity	-0.061204	0.091372	-0.670	0.503
	Resource availability	-0.012200	0.086299	-0.141	0.888
	Shelter availability	-0.064071	0.086126	-0.744	0.457

The table presents fixed-effect estimates, standard errors, z-values, and p-values. Predictor variables include trophic diversity, shelter and substrate diversity and availability, distance from the cave entrance, relative humidity, and air temperature. Statistically significant effects (p < 0.05) are indicated with an asterisk (*)

### Environmental variables determining phylogenetic distance

At the microscale, distance-based redundancy analysis (dbRDA) revealed that variation in phylogenetic distance among communities was significantly associated with distance from the cave entrance (permutational ANOVA: F = 1.11, p = 0.001) and trophic resource diversity (permutational ANOVA: F = 1.04, p = 0.039). No other environmental variables showed significant effects. The overall model was significant (p = 0.004), although it explained only a small fraction of the variation (adjusted R^2^ = 0.001). The first dbRDA axis was significant (p = 0.016) (Fig. S5).

At the mesoscale, dbRDA revealed that variation in phylogenetic distance among communities was significantly associated with distance from the cave entrance (permutational ANOVA: F = 1.51, p = 0.003). No other environmental variables showed significant effects, although relative humidity exhibited a marginal trend (p = 0.061). The first dbRDA axis was significant (p = 0.021). The overall model was significant (p = 0.003), although the proportion of explained variation was low (adjusted R^2^ = 0.011), suggesting that additional unmeasured factors also contribute to structuring phylogenetic community patterns (Fig. S5).

**Fig. 5:**
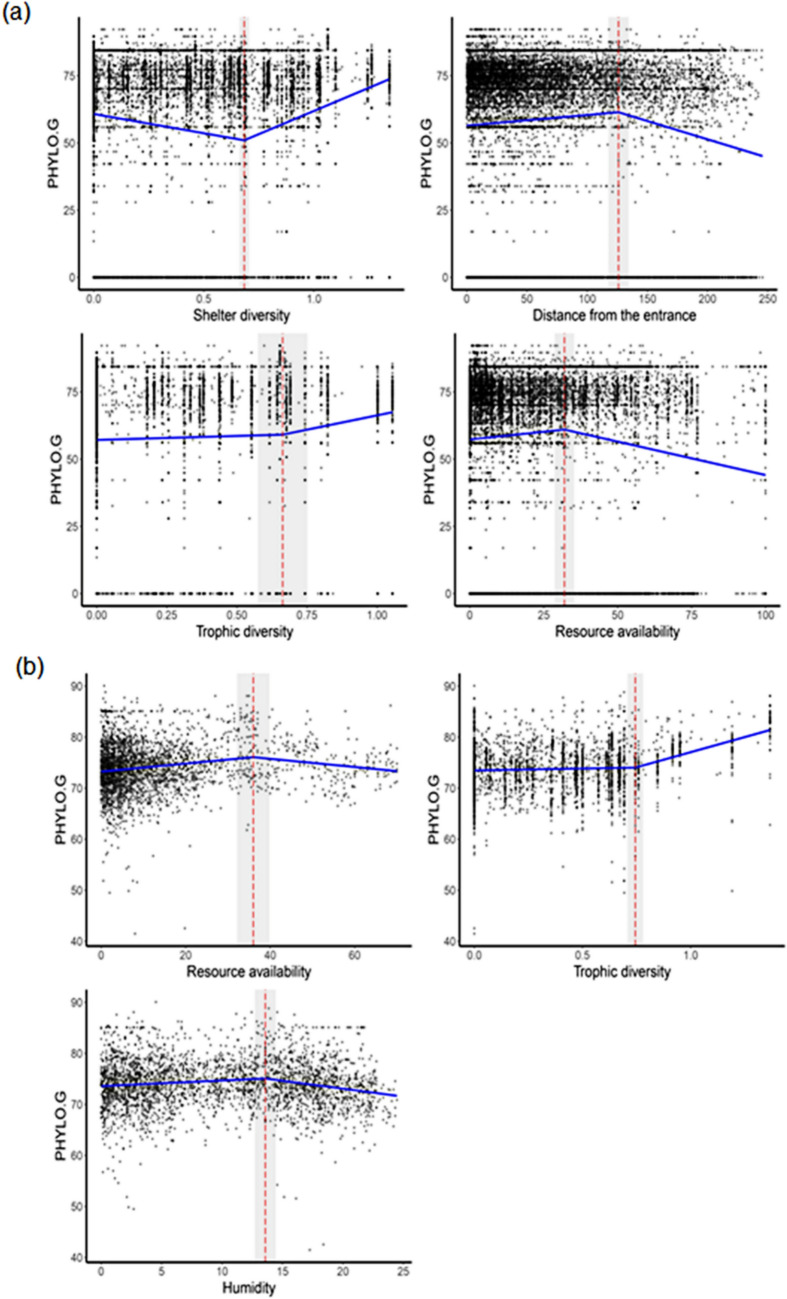
The thresholds in the relationship between phylogenetic distance and significant predictor variables. (**A**) Microscale, (**B**) Mesoscale. The red dashed vertical lines indicate significant change points (p < 0.05), while the shaded gray area represents the standard error around the estimate. Black points represent pairs of communities

At the microscale, exploratory analyses based on generalized linear models and segmented regression indicated that differences in shelter diversity and trophic resource diversity were associated with variation in phylogenetic distance among invertebrate communities. In general, greater differences in these variables were associated with higher phylogenetic distances between community pairs. Differences in trophic resource availability indicated a potential threshold, with intermediate differences associated with higher phylogenetic distances, followed by a decline at higher levels. Similarly, greater differences along the entrance–interior gradient were associated with lower phylogenetic distances, suggesting that communities differing more strongly in their position along this gradient may exhibit reduced phylogenetic differentiation (Fig. 5).

At the mesoscale, exploratory analyses indicated patterns consistent with those observed at the microscale, with differences in trophic diversity and resource availability associated with variation in phylogenetic distance. Differences in relative humidity also indicated a potential threshold, with intermediate differences associated with higher phylogenetic distances between communities. No other environmental variables showed consistent associations with phylogenetic distance. In all cases, segmented regression models provided a better fit than linear models, suggesting the presence of potential ecological thresholds. Full model results are presented in Table 3.

**Table 3:** Segmented regression model analyses evaluating the relationship between phylogenetic distance (distinctness) and different predictive variables at the micro (quadrat) and mesoscales (sectors). Values in bold indicate statistically sgnificant results (p < 0.05)

Spatial scale	Predictor variables	Estimate	Adjust R^2^	Pseudo-F	p-value
	DE	0.029249	0.0002	1.418	0.108
	**TRD**	**11.1025**	**0.0123**	**79.936**	0.0221*
	GSD	1.09008	0.0002	1.33308	0.94
**Meso**	**HUM**	**-0.41853**	**0.003**	**18.8689**	0.0002*
**3 × 10 m**	SHD	-1265.0290	0.0008	5.27062	0.948
	SHA	-0.05151	0.0009	5.64207	0.207
	**TRA**	**-0.15908**	**0.004**	**24.9079**	3.23E-14*
	**DE**	** − 0.175790**	**0.0001**	**23.4017**	2.68E-06*
	**TRD**	**2.9702**	**0.0001**	**12.6473**	0.044*
**Micro**	GSD	0.3446	0.0001	10.0541	0.73
**1 × 1 m**	**SHD**	**-14.2981**	**0.001**	**87.4273**	2.00E-16*
	SHA	-0.004718	0.0004	10.9461	0.637
	**TRA**	**-0.36424**	**0.0004**	**10.5011**	0.0003*

Entrance distance (DE), Trophic resource diversity (TRD), Temperature (TEP), General substrate Diversity (GSD), Humidity (HUM), Diversity Shelter (SHD), Shelter Availability (SHA), Trophic Resource Availability (TRA)

Because these analyses based on generalized linear models and segmented regression rely on pairwise comparisons between communities, involving non-independent observations, their results should be interpreted with caution and considered primarily as indicative of general patterns and potential ecological thresholds, rather than as definitive inferential evidence.

## Discussion

### Spatial variations in faunal similarity

When examining patterns at both micro- and mesoscale levels, significant differences in faunal composition were observed only between two adjacentes limestones belts. Notably, these two regions, despite being geographically closer, exhibited greater compositional dissimilarity than either did in relation to the more geographically distant Itaiacoca region. This result contrasts with findings from previous studies, which have generally suggested that increasing geographic distance between caves is associated with greater faunal differentiation (Souza-Silva et al. 2020; Sovie et al. 2022). However, the two spatially nearest regions (Capiru and Votuverava) demonstrated the highest degree of faunal dissimilarity.

One plausible explanation lies in the underlying lithology and geological history of these formations. Although the Capiru and Itaiacoca regions are geographically distant, they are both predominantly composed of dolomitic rocks, which share similar chemical properties and occupy the same stratigraphic position. In contrast, the Votuverava Formation is geologically younger and composed mainly of calcitic limestones (Marini et al. 1967; Fiori 1992). Previous studies in Brazil have demonstrated that caves formed in different lithotypes can harbor markedly distinct subterranean faunas (Souza-Silva et al. 2011, 2020). In addition, recent research has highlighted that distinct lithologies may present variations in sedimentary structures and cave morphological features, which directly influence the availability of microhabitats and, consequently, the composition of subterranean fauna (Souza-Silva et al. 2011; Vaccarelli et al. 2023; Pacheco et al. 2025), supporting the hypothesis that lithological and paleogeographic factors are major drivers of faunal differentiation.

These findings underscore that even among geographically proximate caves, invertebrate communities can exhibit substantial variation in species composition, corroborating previous studies (Simões et al. 2015; Zagmajster et al. 2018; Mammola et al. 2020a, b; Sovie et al. 2022). Such differences are primarily driven by fine scale environmental conditions, particularly substrate characteristics and microclimatic conditions, which generate highly heterogeneous microhabitats within caves. This ecological heterogeneity plays a key role in shaping invertebrate, community, and distribution patterns, and contributes to reduced faunal similarity between adjacent caves (Pellegrini et al. 2016; Pacheco et al. 2020; Mammola et al. 2020a, b; Souza-Silva et al. 2020).

At the mesoscale, temperature variation emerged, alongside substrate diversity, as a significant factor influencing faunal composition. Species adapted to the stable conditions characteristic of deeper cave zones generally exhibit narrow thermal tolerances and are particularly sensitive to fluctuations in temperature (Souza-Silva et al. 2021; Colado et al. 2022). These taxa benefit from the relative thermal stability of aphotic zones, in contrast to species inhabiting entrance regions, which are exposed to more variable environmental conditions (Colado et al. 2022). Comparable patterns have been reported in karst systems worldwide, where cave temperature and thermal stability are consistently recognized as key determinants of invertebrate community structure. For instance, in the Pyrenees, beetles of the family Leiodidae were strongly associated with microhabitats offering greater climatic stability (Colado et al. 2022). Similarly, Mammola and Isaia (2018) demonstrated that even minor temperature shifts were sufficient to alter the composition of spider assemblages in Italian caves. More recently, showed that the functional composition of European cave spider communities is also modulated by environmental gradients, including temperature variation, further reinforcing the pivotal role of this factor in subterranean community assembly processes (Mammola et al. 2024).

At the microscale, distance from the cave entrance and distance among caves emerged as significant predictors of faunal composition. The environmental gradient extending from the entrance to the deeper zones of a cave is well-documented in the literature (Kozel et al. 2019; Vaz et al. 2025; Da Rocha Melo et al. 2025a). This gradient gives rise to distinct microhabitats that support different species and facilitate a variety of ecological interactions throughout the subterranean environment. Deeper cave zones, however, tend to be more environmentally homogeneous and restrictive, presenting harsher conditions that limit the diversity and distribution of many taxa (Prous et al. 2015; Mammola and Isaia 2018; Lunghi and Manenti 2020). Although the proportion of explained variance was relatively modest at both scales, this outcome is consistent with findings from other studies of subterranean communities and reflects the inherent complexity of ecological processes as well as the influence of unmeasured factors (Pacheco et al. 2025; Junta et al. 2025; da Rocha Melo et al. 2025a). Accordingly, low R^2^ values should not be interpreted as a lack of explanatory power, but rather as evidence of the multifactorial nature of community structuring in cave ecosystems.

Additionally, it is essential to consider the anthropogenic pressures that affect subterranean systems. Many of the caves in the Capiru region are located near active mining areas, which are known to cause a range of negative effects on subterranean environments (Donato et al. 2014). Mining activities can lead to significant alterations in surface ecosystems, including vegetation loss, hydrological disruption, and soil instability, which can directly or indirectly affect the biological communities within adjacent caves (Cardoso et al. 2022). These impacts may result in changes in species richness, composition, and ecological interactions, particularly in caves that serve as critical refuges for specialized or sensitive species (Tscharntke et al. 2002; De Fraga et al. 2023).

### Influence of environmental variables on species richness

Species richness in cave ecosystems is influenced by multiple factors, including the availability of organic resources, substrate heterogeneity, and microclimatic conditions (Ferreira et al. 2007; Tobin et al. 2013; Pellegrini et al. 2016; Pacheco et al. 2020; Souza-Silva et al. 2021). At the microscale, higher resource availability was associated with increased species richness, as the accumulation of organic matter promotes the colonization and establishment of invertebrates, thereby enhancing local biodiversity (Schneider et al. 2011). Moreover, resource diversity supports the formation of more complex trophic networks, which sustain richer communities (Moore et al. 2004; Venarsky and Huntsman 2018).

However, substrate availability tends to decrease with increasing distance from the cave entrance, resulting in lower microhabitat heterogeneity (Prous et al. 2015; Souza-Silva et al. 2021). This environmental simplification imposes increasingly restrictive conditions that limit species richness but does not solely reflect a reduction in resource quantity. Rather, it represents a combined effect of oligotrophy, high environmental stability, and reduced structural diversity (Da Rocha Melo et al. 2025a). Consequently, trogloxenes and troglophiles are primarily concentrated in entrance and twilight zones, where the greater diversity and availability of substrates favor their persistence, whereas troglobites, being more specialized, predominate in deeper regions of the cave, which are characterized by more stable yet more restrictive environmental conditions for other species (Culver and Pipan 2019; Souza-Silva et al. 2021).

### Influence of environmental variables on phylogenetic distance

Distance from the cave entrance significantly influenced the phylogenetic structure of invertebrate communities. This result suggests that the restrictive conditions of inner cave zones, such as absence of light, limited organic input, reduced habitat heterogeneity, and higher humidity, not only shape species composition and richness but also affect the phylogenetic organization of communities (Prous et al. 2015; Souza-Silva et al. 2021). Under these stable yet limiting conditions, highly specialized taxa tend to predominate (Sket 2008), resulting in communities composed of more closely related lineages.

Trophic resource diversity also contributed to the phylogenetic structuring of communities. In contrast, trophic resource availability was not a consistent predictor across all analyzed scales, particularly at the mesoscale, suggesting that oligotrophy (although a defining feature of deeper cave environments) does not operate in isolation as the primary driver of phylogenetic distance. Rather, its influence appears to be modulated by resource heterogeneity and microhabitat complexity, potentially following saturation dynamics in which moderate increases in resource availability enhance phylogenetic distance, whereas high resource abundance favors generalist or opportunistic species, thereby reducing evolutionary distinctness (Moore et al. 2004; Schneider et al. 2011). This pattern reinforces that resource diversity exerts a stronger influence on the structure of subterranean communities than resource abundance alone (Souza-Silva et al. 2021; Reis-Venâncio et al. 2024; Sant’Ana Merlo et al. 2026). In this context, some species exhibit well-defined feeding preferences. *Acherontides eleonorae*, for instance, has been consistently recorded in association with guano deposits, together with dipteran larvae and adult beetles of the family Leiodidae, as previously reported by Palacios-Vargas and Gnaspini-Netto (1992). Similarly, isopod species have been found in areas with decaying wood, suggesting a preference for this type of resource, a pattern also observed by Souza-Silva et al. (2021).

Despite the statistical significance of the results, the relatively low explanatory power of the tested variables indicates that a substantial proportion of the variation in the phylogenetic structure of cave invertebrate communities remains unexplained. This pattern suggests that, although the measured environmental factors act as relevant ecological filters, they account for only a limited subset of the processes structuring these communities. In subterranean environments, current knowledge of the mechanisms underlying biological organization remains incomplete, and several studies have similarly reported low explanatory power in their analyses (Souza-Silva et al. 2021; Reis-Venâncio et al. 2022; Pacheco et al. 2025).

In this context, processes that are often not quantified in subterranean ecological studies, such as biotic interactions (e.g., predation and competition), metacommunity dynamics, ecological succession, biological invasions, and stochastic events, may play an important role in community assembly, thereby contributing to the limited variance explained by the models (Da Rocha Melo et al. 2025a). Consequently, low R^2^ values should not be interpreted as a weakness of the results but rather as a reflection of the intrinsic complexity of cave systems, in which multiple processes operate simultaneously across different spatial and temporal scales (Mammola 2019). From this perspective, our findings reinforce the view that the ecological “puzzle” of caves is composed of multiple complementary components, with the physical microhabitat representing only one (albeit a fundamental) piece.

### Final remarks

Our results demonstrate that physical, trophic, and microclimatic factors interact in an integrated manner to structure cave invertebrate communities, with spatial scale emerging as a central component of ecological organization. By integrating environmental and biological data across multiple spatial scales, we show that patterns of cave biodiversity are primarily governed by environmental filtering linked to habitat heterogeneity and resource distribution. These findings advance the understanding of community assembly processes in cave ecosystems and underscore the importance of scale-explicit approaches in subterranean ecology.

From a conservation perspective, our results indicate that effective cave protection requires both the preservation of entire cave systems and the maintenance of fine-scale environmental features that sustain key ecological processes. Nevertheless, this study has some limitations, including restricted taxonomic resolution, the absence of molecular data to resolve evolutionary relationships, and the lack of systematic sampling of cave walls and ceilings. These constraints may limit the generality of our conclusions and highlight the need for complementary and comparative studies encompassing broader geographic, ecological, and taxonomic scopes.

## Supplementary Information

Below is the link to the electronic supplementary material.

Supplementary file1

Supplementary file2

Supplementary file3

## Data Availability

Datasets generated or analyzed in this study are available from the corresponding author on reasonable request.
