# Supplementary material for: Unveiling the microhabitat puzzle: how spatial heterogeneity shapes cave invertebrate biodiversity across scales
Source: Oecologia. 2026 Jun 29;208(7):89. doi: 10.1007/s00442-026-05928-z (PMC13314787; doi:10.1007/s00442-026-05928-z)
Supplement: Supplementary file 1 — Supplementary file1 [file 442_2026_5928_MOESM1_ESM.docx]

**Unveiling the microhabitat puzzle: how spatial heterogeneity shapes cave invertebrate biodiversity across scales**

Leandro Mata da Rocha Melo^1,2*^, Rodrigo Lopes Ferreira^1,2^, Marconi Souza Silva^1,2^

^1^Centro de Estudos em Biologia Subterrânea, Departamento de Ecologia e Conservação, Instituto de Ciências Naturais, Universidade Federal de Lavras, Campus Universitário, P.O. Box 3037, Lavras CEP 37200-000, Minas Gerais, Brasil

^2^Programa de Pós-Graduação em Ecologia Aplicada, Universidade Federal de Lavras, Lavras CEP 37200-000, Minas Gerais, Brasil

e-mail:*[Leandro.matabio@gmail.com](mailto:Leandro.matabio@gmail.com)

Telephone: +55 37 998070126

**Supplementary table I**. List of substrates found in the sampled caves, the abbreviations used, and their classification for analysis. Hardpan = Compacted clay. W=Width

| Substrates type | Grain size | Abbreviation | Categories |
| --- | --- | --- | --- |
| Waterbody | Does not apply | WB | General |
| Sand | (2-0,06 mm) | SND | General |
| Silt/clay/mud | Does not apply | SCM | General |
| Speleothem | Does not apply | SP | General |
| Hardpan | Does not apply | HP | General |
| Inorganic substrate | Does not apply | IS | General |
| Dropping of Speleothem | Does not apply | DS | General |
| Termite mounds | Does not apply | TM | General l |
| Shrinkage crack | Does not apply | SC | Shelter |
| Smooth Rock floor | Does not apply | SR | Shelter |
| rough rock floor | Does not apply | RR | Shelter |
| Big boulder | 4000-250mm | BB | Shelter |
| Small Block | 250-64mm | SB | Shelter |
| Gravel | 64-2mm | GR | Shelter |
| Snail Shell | Does not apply | SS | Shelter |
| Guano | Does not apply | GA | Trophic resource |
| Feces | Does not apply | FC | Trophic resource |
| Roots | Does not apply | RT | Trophic resource |
| Cryptogams | Does not apply | CR | Trophic resource |
| Phanerogams | Does not apply | PHA | Trophic resource |
| Algae | Does not apply | AL | Trophic resource |
| Actinomycetes | Does not apply | ACT | Trophic resource |
| Basidiomycetes | Does not apply | BAS | Trophic resource |
| Animal carcass | Does not apply | AC | Trophic resource |
| Pteridophytes | Does not apply | PT | Trophic resource |
| Leaf litter | >10mm | LFL | Trophic resource /shelter |
| Branches | 250-11mm (w) | BR | Trophic resource /shelter |
| Stem | >250mm (w) | ST | Trophic resource /shelter |


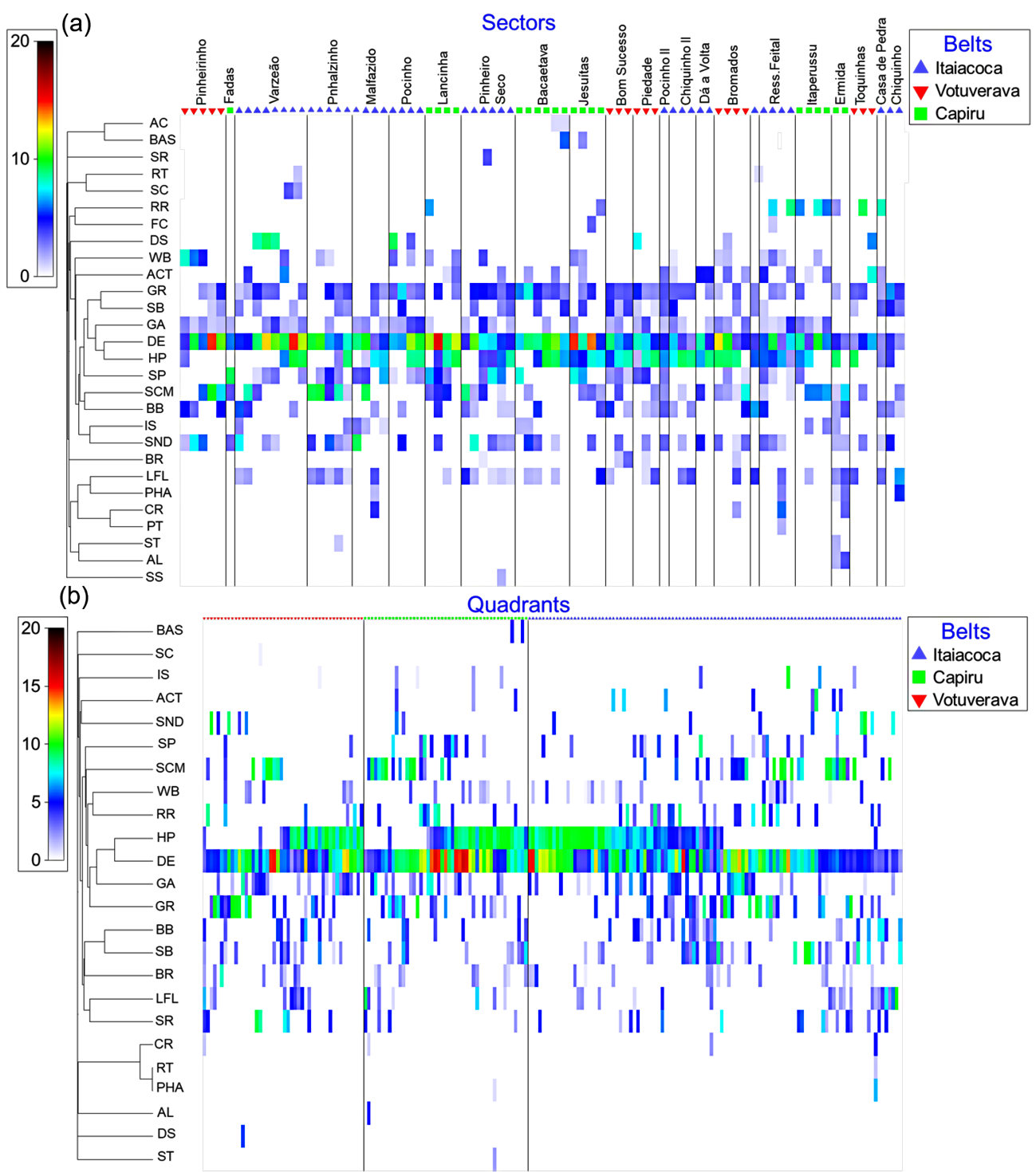


**Figure S1**. Variations in the distribution and percentage of area occupied (values from 0 to 20) by components of the substrate on the floor of the caves and their associations with the distance from the entrance (DE) in the sectors (a) and quadrats (b). The dendrogram on the left groups the sample units according to the Whittaker association index. Substrates type: WB-Waterbody; SND-Sand; SCM-Silt/Clay/Mud; SP-Speleothem; HP-Hardpan; IS- Inorganic substrate; DS- Dropping of Speleothem; TM-Termite mounds; SC-Shrinkage crack; SR- Smooth Rock floor; RR- Rough rock floor; BB-Big Boulder; CBD- Small Block; GR-Gravel; CON- Snail Shell; GA-Guano; FC-Feces; RT-Roots; CR-Cryptogams; PHA- Phanerogams; AL-Algae; ACT- Actinomycetes; BAS-Basidiomycetes; AC- Animal carcass; PT- Pteridophytes; LFL- Leaf litter; BR- Branches; ST-Stem.


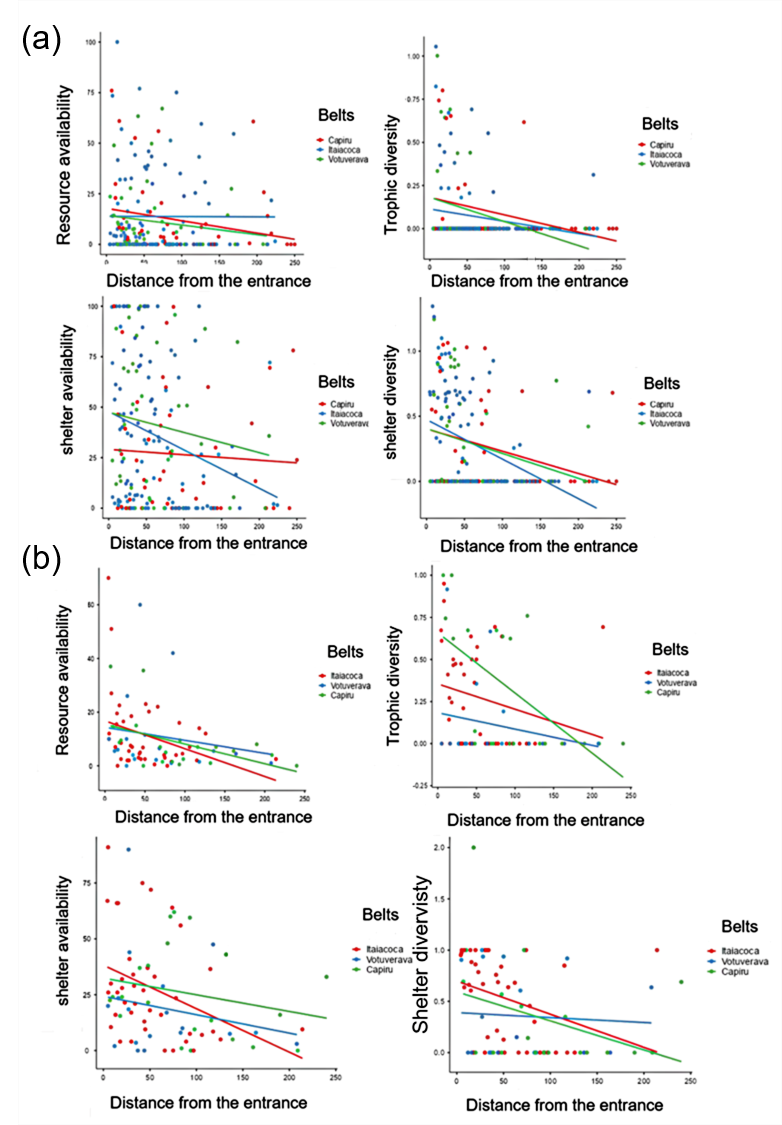


**Figure S2**. Relationship between diversity and availability of substrates, shelters, and trophic resources with the distance from the entrance in quadrats (a) and sectors (b).


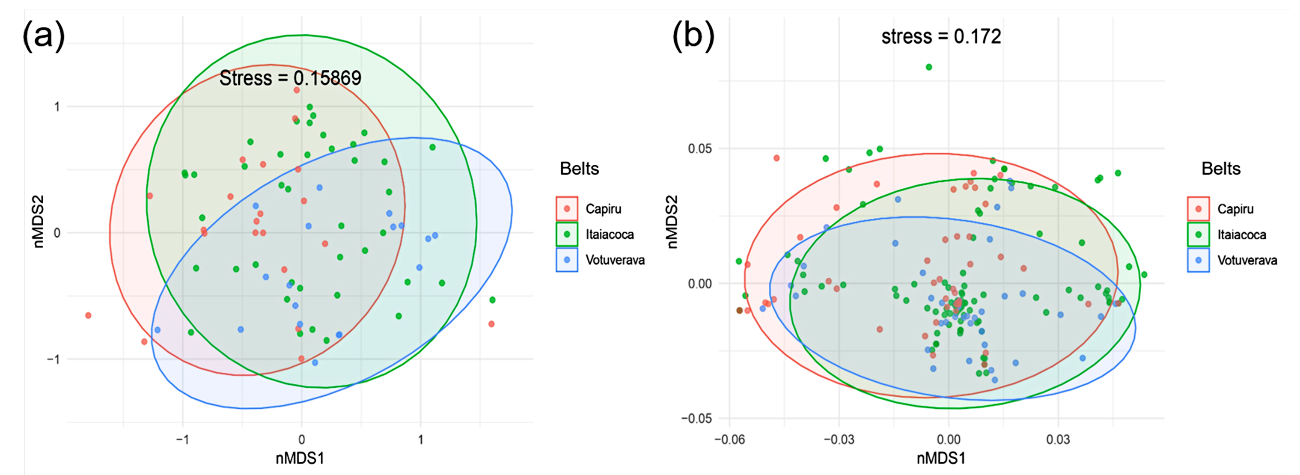


**Figure S3.** Multidimensional scaling (nMDS) shows the dispersion of fauna similarity in sectors (a) and quadrats (b). The dispersion of the points indicates the variation in the fauna composition within each limestone region.


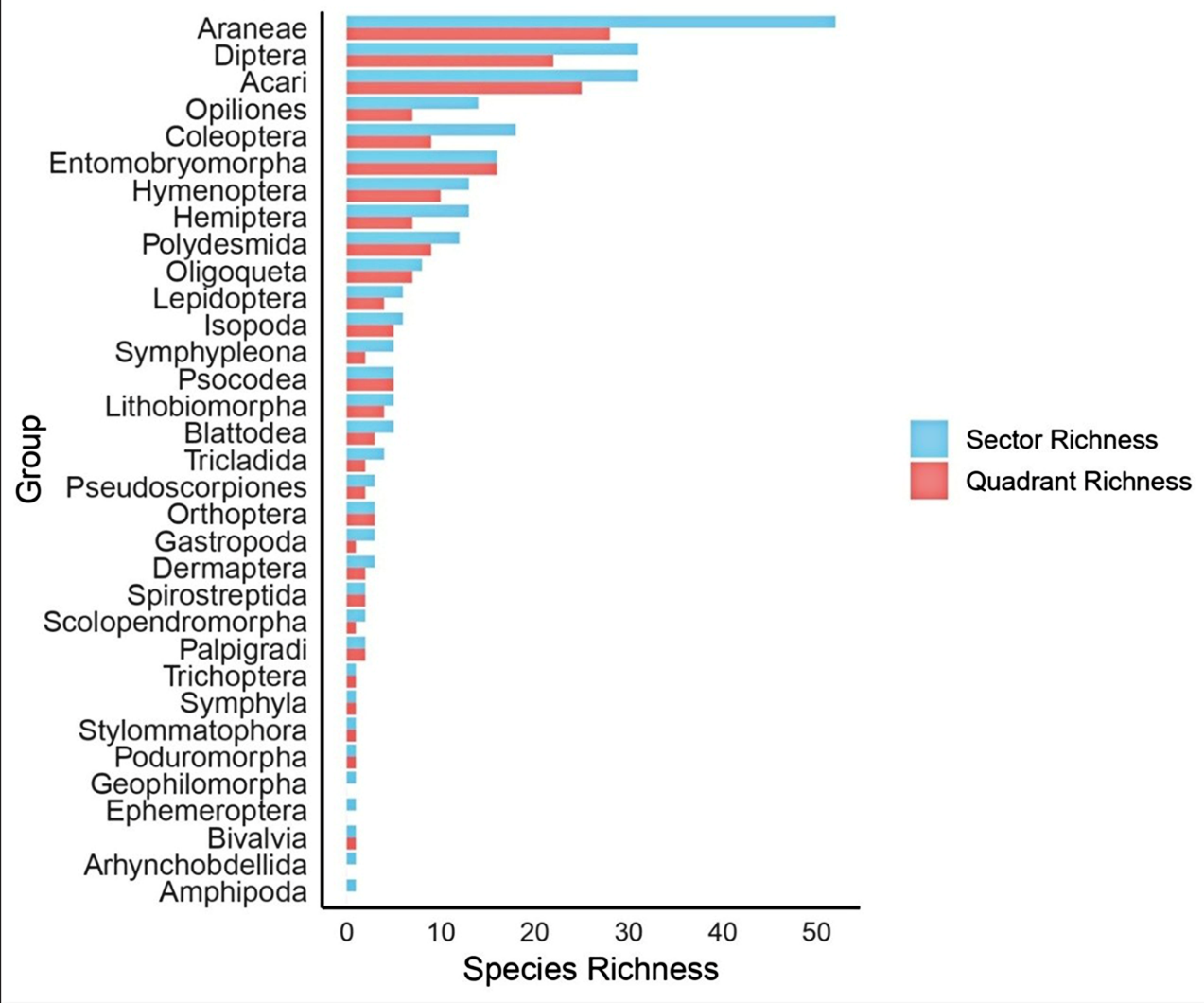


**Figure S4**. The richness of invertebrates in different taxa was observed in 23 caves in the state of Paraná. The blue lines represent the richness in the sectors, and the red lines represent the richness in the quadrats.


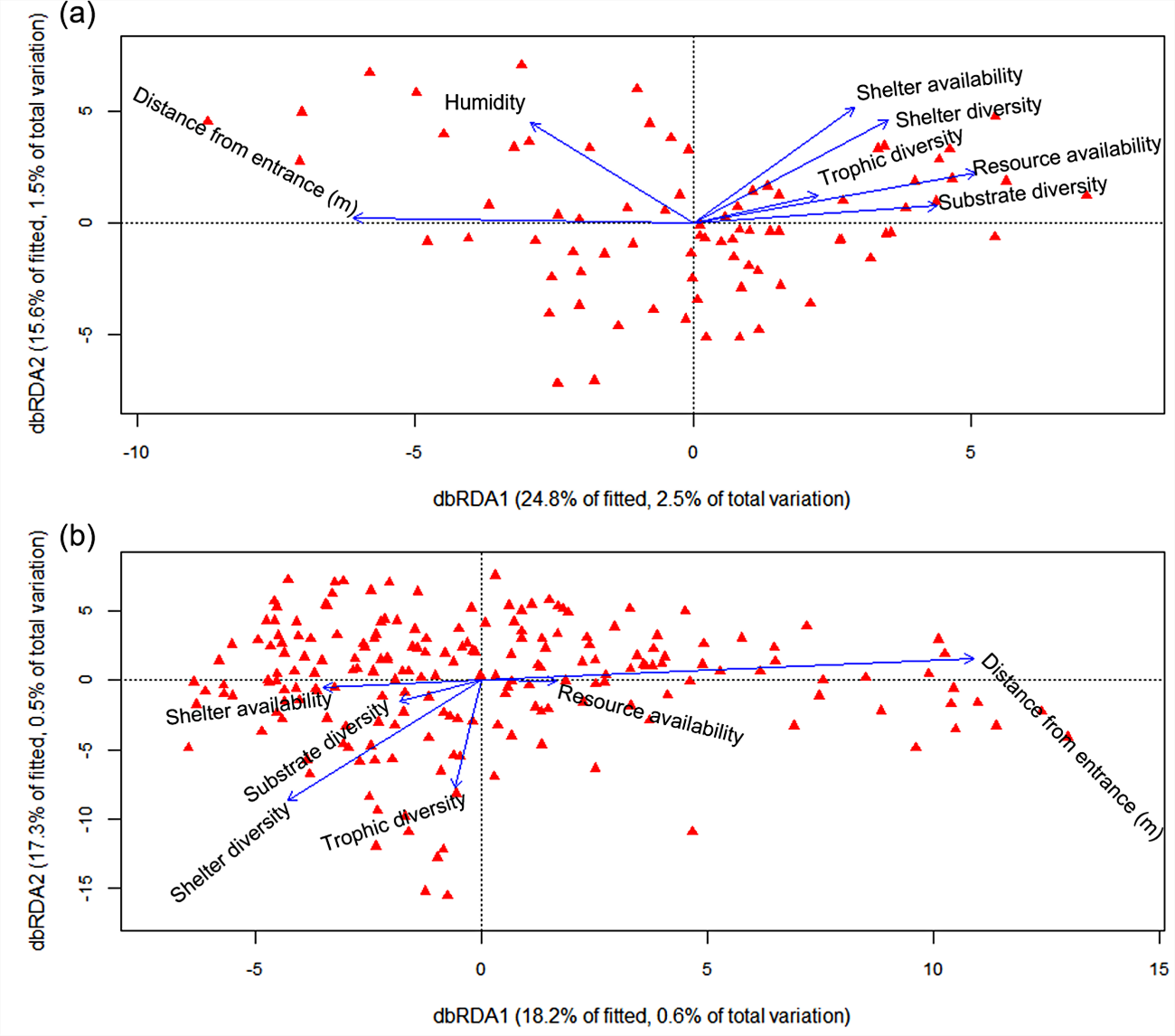


**Figure S5.** Distance-based redundancy analysis (dbRDA) illustrating the relationships between community phylogenetic structure and spatial and environmental gradients at the (a) micro-scale and (b) meso-scale. Red triangles represent sampling units. Blue arrows indicate spatial and environmental predictors, with arrow length and direction proportional to the strength and orientation of their correlations with the ordination axes.
